# A Rare Case of Non-cirrhotic Hepatocellular Carcinoma in a Female Adolescent: Diagnostic and Multimodal Treatment Challenges in a Low-Resource Setting

**DOI:** 10.7759/cureus.99925

**Published:** 2025-12-23

**Authors:** Yatrasana Singh, Barry Raghunanan, Ravi Maharaj

**Affiliations:** 1 General Surgery, University of the West Indies, St. Augustine Campus, Champ Fleurs, TTO; 2 Surgery, University of the West Indies, St. Augustine Campus, Champ Fleurs, TTO

**Keywords:** hepatoblasoma, hepatocellular carcinoma (hcc), non-cirrhotic hepatocellular, right hepatectomy, sorafenib

## Abstract

Hepatocellular carcinoma (HCC) is rare in children and adolescents, particularly in non-cirrhotic livers. Pediatric HCC often presents with aggressive behaviour and requires individualized, multidisciplinary care. Liver transplantation is a potential curative option, but this procedure is not readily accessible in many regions. In this case, a 15-year-old female patient presented with vague right-sided abdominal pain, fatigue, and abdominal distension. Imaging revealed a large heterogeneous hepatic mass with right portal vein thrombosis and mass effect on the inferior vena cava. Laboratory investigations revealed elevated transaminases and negative viral hepatitis markers. Histology was suggestive of HCC with possible hepatoblastoma features. Despite the tumour’s size potentially qualifying her for a liver transplant according to the Milan and University of California, San Francisco criteria, we opted for neoadjuvant chemotherapy and a resectional approach due to inherent limitations. She underwent four cycles of neoadjuvant sorafenib-based chemotherapy, followed by extended right hepatectomy and right hemi-diaphragmatic resection. A postoperative bile duct injury was successfully managed with the endoscopic retrograde cholangiopancreatography rendezvous technique for biliary stenting. In settings without readily accessible transplant capability, aggressive multimodal therapy including neoadjuvant chemotherapy and liver resection can offer curative potential. This case highlights the importance of adapting treatment strategies to available resources while maintaining oncologic intent.

## Introduction

Hepatocellular carcinoma (HCC) in children is an uncommon but highly aggressive malignancy, accounting for ~ 0.7% of all childhood malignancies worldwide [1]. It represents the second most common primary malignant liver tumour in children, accounting for ~20-30% of all paediatric primary hepatic malignancies, after hepatoblastoma, which comprises 60-70% of cases [1-3]. Unlike the adult form, which is often linked to cirrhosis and chronic viral hepatitis, paediatric HCC more commonly arises in otherwise normal livers, making early detection challenging and prognosis generally poor [2].

Survival outcomes for paediatric HCC remain significantly poorer than those seen in adults, particularly in advanced disease. The overall five-year survival rate for resectable paediatric HCC ranges between 20-30%, with a five-year survival rate of <30% in advanced paediatric HCC. In contrast, adult HCC has shown improvements in outcomes with surveillance programs and locoregional therapies, leading to a five-year survival rate of 30-40% once diagnosed early [3-4]. There has also been a documented rise in non-viral, metabolic-associated HCC among adolescents, which mirrors the global rise in obesity, metabolic syndrome, and non-alcoholic steatohepatitis (NASH), and so classical imaging and serologic criteria used in adults may not apply [5-7].

Diagnosis is complicated by nonspecific symptoms, overlapping imaging features with other hepatic masses, and variable tumour biology. While transplantation is curative, most low-resource centres lack access, framing this case as representative of global inequality in treatment options and adding another layer of complexity to management. Advances in systemic and targeted therapies have begun to expand options, but outcomes remain largely dependent on resectability and timely intervention [8-10]. 

We report the case of a 15-year-old female adolescent with hepatocellular carcinoma arising in a non-cirrhotic liver, with no known predisposing risk factors. This case underscores the diagnostic challenges and highlights the importance of multimodal therapy in achieving curative outcomes in settings where transplantation is not available.

## Case presentation

A 15-year-old girl presented to the emergency department with right-sided vague abdominal pain, fatigue, and abdominal distension for three months. She was anicteric with no history of blood transfusions or jaundice, and no family members with similar complaints. Antenatal history was unremarkable with negative hepatitis B and C titres in the mother, and the patient received all the scheduled vaccines during infancy. Her laboratory evaluation demonstrated markedly elevated liver enzymes and preserved synthetic function. Key laboratory values are summarized in Table 1. Physical examination revealed anicteric sclera, a palpable liver edge with mild tenderness, and no ascites. 

**Table 1:** Summary of Relevant Laboratory Investigations AST, aspartate aminotransferase; ALT, alanine aminotransferase; ALP, alkaline phosphatase; AFP, alpha-fetoprotein; β-hCG, beta-human chorionic gonadotropin; CEA, carcinoembryonic antigen

Parameter	Result	Reference Range
AST ( U/L)	700	8-33
ALT (U/L)	175	4-36
ALP (U/L)	202	44-147
Total bilirubin (mg/dL)	1.4	0.2-1.0
Direct bilirubin (mg/dL)	0.0	0.0-0.2
Indirect bilirubin (mg/dL)	0.8	0.2-0.8
AFP (NG/mL)	>60,000	0-20
Beta-hCG (mIU/ML)	<2.39	<5 (non-pregnant)
CEA (ng/mL)	1.4	0-3

**Figure 1:**
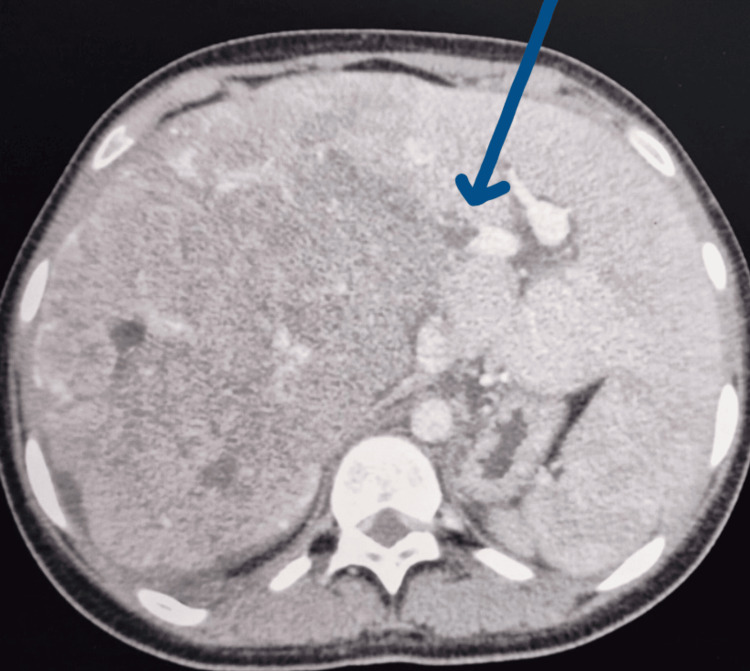
Contrast-enhanced computed tomography showing diffusely enlarged heterogeneous liver involving the whole of the right lobe with peripherally enhancing lobulated components and internal cystic areas with a mass displacing the IVC, and an arrow showing right portal vein thrombus pre-neoadjuvant chemotherapy IVC: inferior vena cava

Contrast-enhanced computed tomography of the abdomen/pelvis revealed a diffusely enlarged heterogeneous liver with peripherally enhancing lobulated components and increased vascularity in the right hepatic lobe. Internal cystic areas were noted, suggestive of necrosis with a large cystic component measuring 6.7cm x 6.8 cm. The liver measured 19.6 cm (longitudinal section). There was no invasion of the anterior abdominal wall. The hepatic mass displaced the inferior vena cava (IVC) to the left with mass effect and compression, measuring 0.3 cm at its narrowest with preserved patency. Right portal vein thrombus was noted with non-opacification of the right portal vein and mild peri-portal oedema, as noted in Figure 1A. A few subcentimetre lymph nodes were noted at the anterior superior aspect of the liver, the largest measuring 0.9 cm with mild abdominal free fluid. The right adrenal gland was not positively identified, and the right kidney was displaced inferiorly. The gallbladder showed no intra- or extra-hepatic duct dilatation. Of note, a whole body FDG PET-CECT scan, although recommended prior to resection of HCC, was not available at this institution.

**Figure 2:**
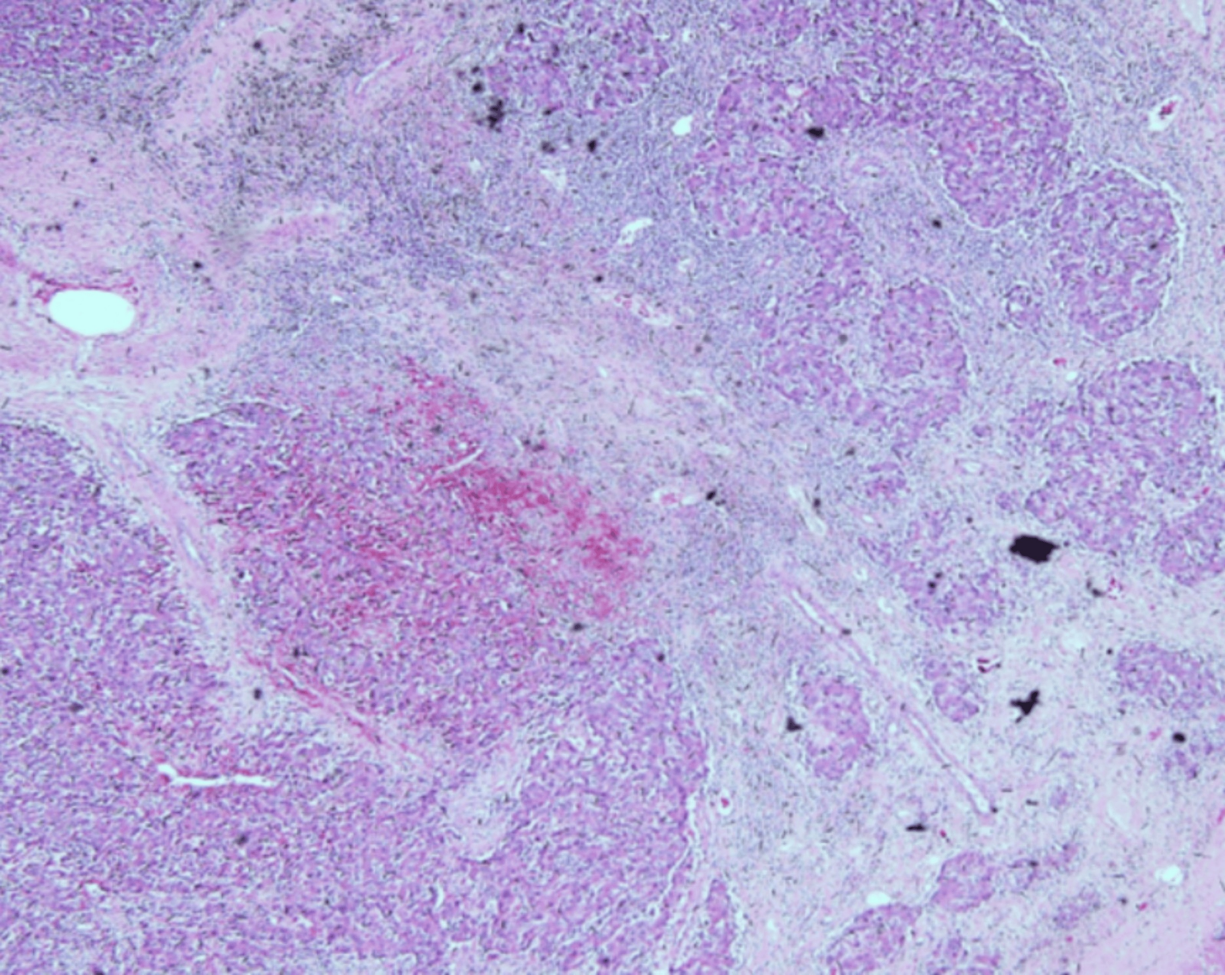
Haematoxylin and eosin-stained x 200 Haematoxylin and eosin staining demonstrating poorly differentiated hepatocellular carcinoma arranged in trabecular and solid sheets. Tumour cells show marked cytologic atypia with prominent nucleoli and focal clear-cell change. Areas of necrosis are present, consistent with high-grade morphology.

**Figure 3:**
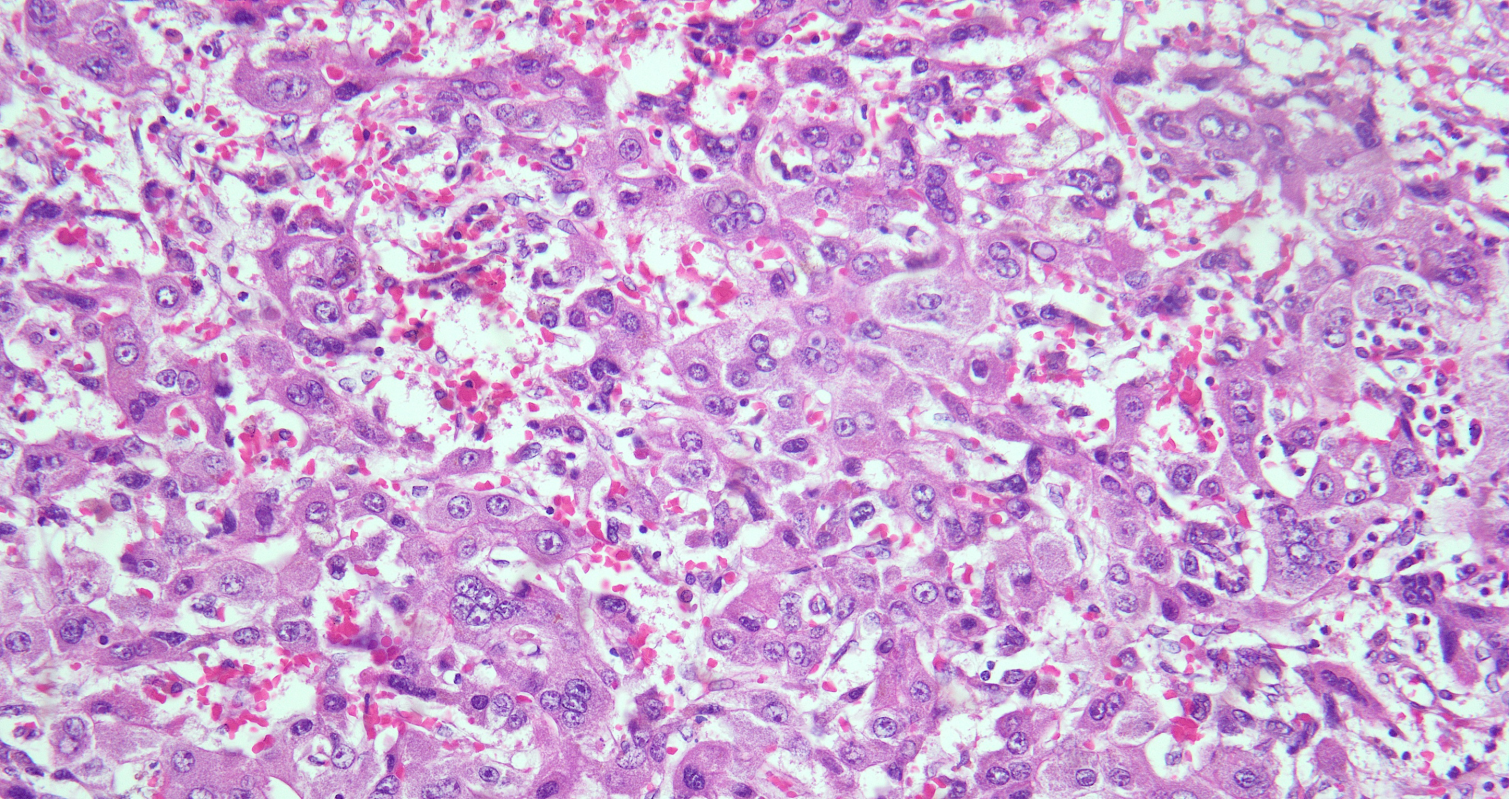
Liver biopsy (H&E stain) - high-power view. H&E stain demonstrating sheets of markedly atypical hepatocellular tumour cells with enlarged pleomorphic nuclei, abundant eosinophilic cytoplasm, and scattered multinucleated giant cells with areas of necrosis consistent with poorly differentiated HCC.

Ultrasound-guided liver biopsy showed fragmented cores of a malignant tumour characterized by a compact and trabecular proliferation of neoplastic cells. These cells exhibited a dimorphism variation with abundant eosinophilic cytoplasm and distinct nucleoli, as shown in Figure 2. The lesser components exhibited clear cell change and moderate nuclear atypia as seen in Figure 3. Focally, there was a proliferation of immature hepatocytes. Bile stasis was noted, but no other inclusions. Immunohistochemistry revealed beta-catenin, diffuse strong reactivity with cytoplasmic localization, moderate reactivity of Glypican-3 (membranous and cytoplasmic), and negative CEA. The conclusion was that the findings were supportive of a hepatocellular neoplasm, not otherwise specified (NOS), with features suggestive of HCC and a possible hepatoblastoma component.

After a multidisciplinary discussion, the patient underwent four cycles of chemotherapy with sorafenib, after which she was radiologically restaged with a CECT of the chest, abdomen, and pelvis, as well as with a liver MRI.

**Figure 4:**
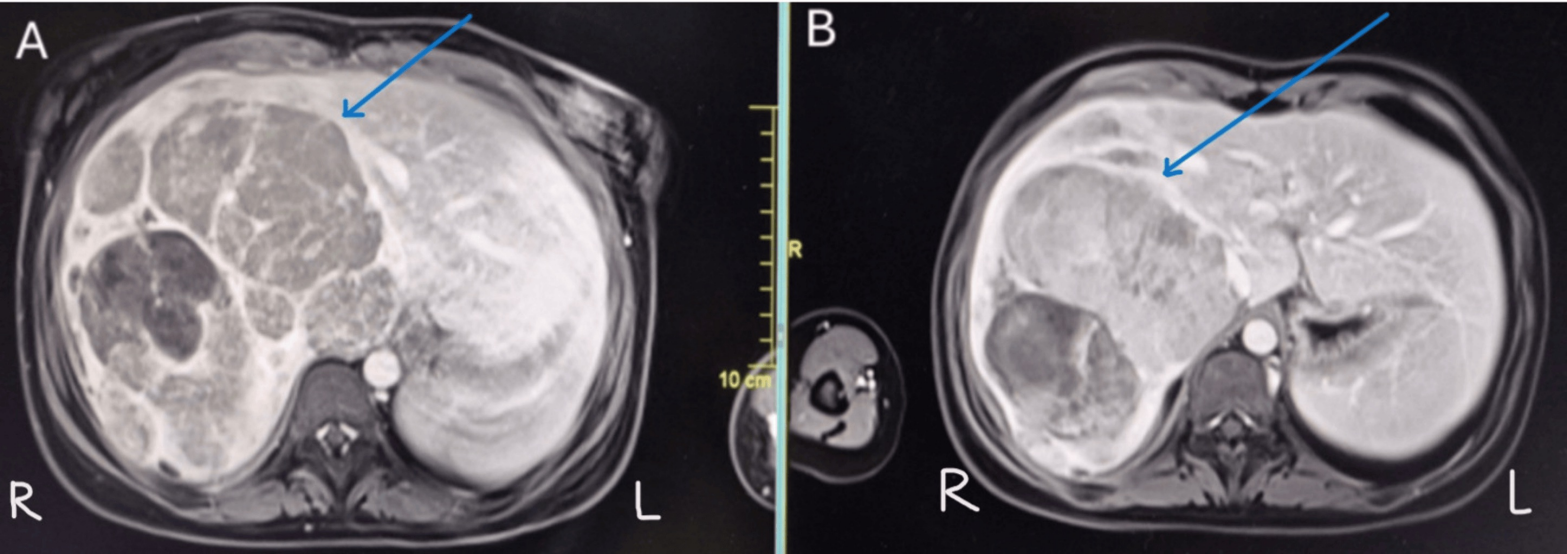
(A) Contrast-enhanced MRI of the liver showing a large, heterogeneous right lobe mass with restricted diffusion and enhancing solid components (arrow); (B) post-neoadjuvant imaging demonstrating normal left lobe of the liver and mild interval tumour shrinkage with preserved left lobe, corresponding to PRETEXT III staging (arrow).

The MRI liver showed a mild interval decrease in size of the ill-defined, lobulated, heterogeneous hepatic mass involving the right side of the liver, measuring 11.1 (transverse section) x 12.3 (anteroposterior) x 15.2 cm (craniocaudal) with no extension of the mass beyond the Glisson capsule. The IVC was compressed with clear fat planes between the liver, and no evidence of thrombus was seen. The right and left portal veins were normal, with no thrombus shown in Figure 4. 

**Figure 5:**
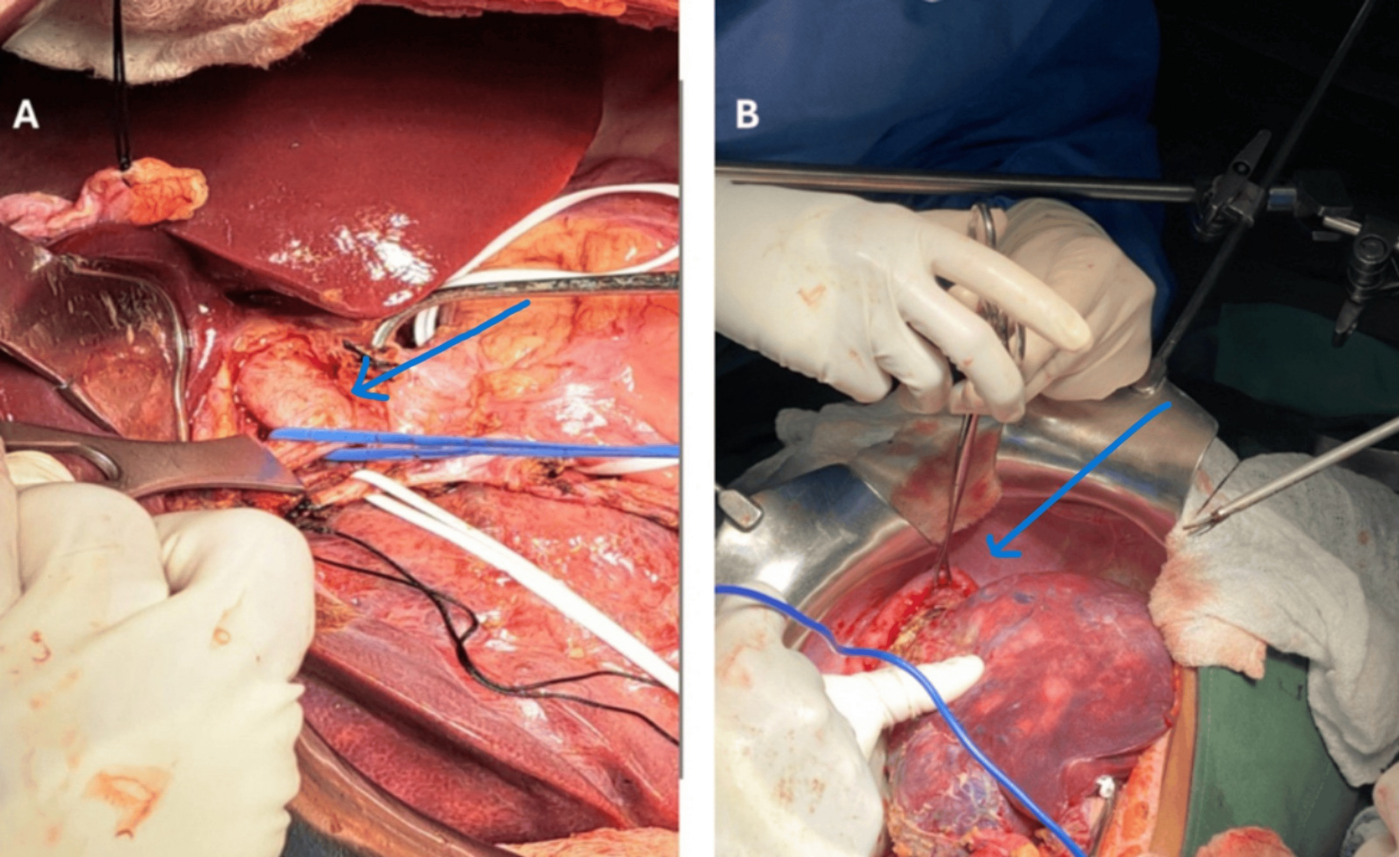
Panel A shows the right portal vein branch indicated by a blue arrow during vascular control prior to parenchymal transection. Panel B demonstrates the right hemidiaphragm (arrow) adherent to the hepatic mass, necessitating en bloc resection of the right diaphragm.

She went on to have an extended right hepatectomy via a chevron incision using intraoperative guidance and a right hemidiaphragm resection that was adherent to the tumour, as seen in Figure 5, followed by primary repair. A ductal injury was diagnosed postoperatively due to increased biliary output, and ERCP and biliary stenting were done via a rendezvous technique. 

Table 2 is a timeline showing imaging and treatment milestones for this case. 

**Table 2:** Summary of the clinical timeline

Time Point	Event
Three months prior to presentation	Onset of vague right-sided abdominal pain, fatigue and abdominal distension
Initial presentation	Emergency department visit; abnormal LFTs and markedly elevated AFP (> 60,000 ng/mL)
Diagnostic work-up	CECT abdomen/pelvis showing a large heterogeneous right lobe hepatic mass with right PV thrombosis and IVC compression
Week 1	Ultrasound-guided liver biopsy performed; histology suggestive of poorly differentiated HCC with hepatoblastoma-like features
Multidisciplinary discussion	Decision for neoadjuvant therapy due to tumour size, vascular involvement and absence of transplant access
Weeks 1-12	Four cycles of sorafenib-based neoadjuvant chemotherapy
Restaging (Post-chemotherapy)	MRI liver showed interval tumour reduction and resolution of portal vein thrombus (PRETEXT III)
Week 13	Extended right hepatectomy with right hemidiaphragm resection
Postoperative course	Bile duct injury diagnosed due to high-output biliary drainage
Postoperative day 7-10	ERCP with rendezvous technique- successful biliary stenting
Follow-up	Completion of adjuvant sorafenib; patient remains disease-free up to date

## Discussion

HCC in paediatric patients is a rare but aggressive malignancy; unlike adult HCC, which is strongly associated with chronic liver disease, approximately 70% of paediatric HCC cases occur in a non-cirrhotic liver and are often diagnosed at an advanced stage with either locally invasive or metastatic disease [3]. Risk factors in children include perinatally acquired hepatitis B virus (HBV) infection, hereditary tyrosinemia, progressive familial intrahepatic cholestasis, Alagille syndrome, congenital portosystemic shunts, and metabolic disorders such as glycogen storage disease [4]. Notably, the male-to-female ratio ranges from 2:1 to 4:1, with oestrogen theorized to play a protective role by reducing viral replication and hepatic inflammation [3].

Our patient, a 15-year-old girl, presented outside of these common demographic and clinical profiles, with no prior liver disease or known risk factors, an uncommon scenario. Notably, fibrolamellar HCC (FL-HCC), a subtype more common in the younger female population, often occurs in the absence of underlying liver disease [4]. FL-HCC tends to show better survival outcomes (five-year survival of 57%) compared to the conventional subtype, which has a five-year survival rate of 28% [3-4].

Alpha-fetoprotein (AFP) is considered a cornerstone biomarker in HCC diagnosis. It is elevated in approximately 50-65% of paediatric HCC cases and has diagnostic as well as prognostic implications. This patient's markedly elevated AFP level aligns with reports showing median AFP values between 20,000 and 100,000 ng/mL in paediatric cohorts, where higher levels are associated with a high tumour burden [5]. However, its sensitivity is limited, particularly in fibrolamellar variants where levels may remain normal. Additional histologic markers such as Glypican-3, Arginase-1, and HepPar-1 help refine diagnostic accuracy, particularly in poorly differentiated tumours [6].

Histologically, paediatric HCC demonstrates variable differentiation and can present with large pleomorphic cells, multinucleated tumour giant cells, and a fibrotic stroma, especially in the fibrolamellar subtype. Given the potential overlap with hepatoblastoma and other liver masses such as focal nodular hyperplasia, hepatic adenoma, or embryonal sarcoma, histopathological and immunohistochemical confirmation remains essential. The histologic features in this case required consideration of several differential diagnoses, including hepatoblastoma, fibrolamellar carcinoma (FL-HCC), and benign hepatocellular proliferations. Hepatoblastoma was unlikely given the patient's age and absence of fetal epithelial patterns, as well as a lack of mesenchymal elements. FL-HCC can be excluded by the absence of lamellar fibrosis, oncocytic cytoplasm, and its characteristic DNAJB1-PRKACA fusion. Benign entities such as focal nodular hyperplasia can be ruled out due to marked atypia with necrosis and disorganized growth [2, 6, 7].

Although molecular profiling (e.g., CTNNB1 or TERT promoter mutations) was not available, the tumour's strong beta-catenin and Glypican-3 expression supported a diagnosis of HCC. The WHO 2022 classification recognizes a spectrum of hepatoblastoma-HCC overlap lesions, also termed transitional liver cell tumour (TLCT), which share mixed features; however, the overall morphological features and immunophenotype in our case were most consistent with those of a poorly differentiated HCC [2, 7].

The absence of chronic liver disease, normal bilirubin levels, and equivocal AFP levels can complicate diagnosis. Our patient had mildly elevated liver enzymes and elevated AFP levels; however, a normal AFP level is not an uncommon finding seen in fibrolamellar or poorly differentiated tumours. Histology confirmed a malignant hepatic neoplasm with both eosinophilic and clear cell changes, suggesting HCC with hepatoblastoma-like features. Immunohistochemistry findings (positive beta-catenin and glypican-3, negative CEA) supported the diagnosis of a hepatocellular neoplasm of uncertain subtype, with possible mixed features [6]. This overlap has been increasingly reported in paediatric populations [7-8].

Imaging plays a central role in guiding surgical decision-making. Initially, the SIOPEL-defined Pre-treatment Extent of Disease (PRETEXT) system is the most widely used paediatric liver tumour staging tool. Prognostic factors include tumour multifocality, vascular invasion, metastatic spread (commonly to lungs), and performance of surgical resection [7]. Multifocal disease significantly reduces the five-year survival rate (0-26%) compared to unifocal tumours (21-70%). The radiological and histological findings suggested aggressive tumour biology. While FDG-PET is useful in adults for detecting extrahepatic spread, it was not available in our low-resource setting. Using retrospective radiologic assessment based on the PRETEXT criteria, the patient's tumour was staged as PRETEXT III, consistent with involvement of three contiguous sectors and preservation of the left lateral sector [7, 8, 10]. 

Liver transplantation is a potentially curative choice for paediatric HCC, particularly in cases where the tumour is unresectable or associated with poor hepatic reserve. Several transplant selection criteria have been developed in adult HCC, most notably the Milan criteria (single lesion ≤5 cm or up to three lesions each ≤3 cm, no vascular invasion or extrahepatic spread) and the UCSF criteria (single lesion ≤6.5 cm or up to three lesions with the largest ≤4.5 cm and total tumour diameter ≤8 cm). While not developed specifically for children, these frameworks are often applied due to the lack of paediatric-specific criteria [7, 10].

Initial CECT findings, demonstrating right portal vein thrombosis, IVC compression, and a large heterogeneous mass, supported the decision to begin neoadjuvant therapy rather than attempt upfront resection. Liver transplantation was also not available at our institution, and regional access is limited by resource constraints, donor shortages, and lack of paediatric transplant programs. 

Following sorafenib-based chemotherapy, interval MRI showed a measurable reduction in tumour size, resolution of portal vein thrombus, and preservation of the left lateral sector, effectively shifting the tumour to a resectable PRETEXT II category. These changes allowed the surgical team to plan a safe extended right hepatectomy with an anticipated adequate future liver remnant. This case reflects the reality in many low-resource settings, where clinical decision-making must balance optimal oncologic management with infrastructure limitations. Importantly, successful resection following tumour downstaging with sorafenib underscores that even in resource-constrained environments, curative outcomes are possible with timely intervention and multidisciplinary care.

For early-stage (PRETEXT I-II) tumours, complete resection alone or with adjuvant chemotherapy (e.g., cisplatin and doxorubicin-PLADO regimen) yields favourable outcomes, with survival rates reaching up to 90% [7-8]. In unresectable cases, neoadjuvant chemotherapy has been used to downstage tumours, with some success in making patients eligible for resection or transplantation [9]. In our case, neoadjuvant chemotherapy was effective in facilitating surgical intervention. The radiologic response following neoadjuvant sorafenib is consistent with small paediatric series in which 10-30% tumour size reduction has been documented, enabling resectability in previously inoperable cases [8-9]. 

Recent data have also shown promising outcomes with targeted therapies such as sorafenib, especially in combination with chemotherapy. In a multi-centre study of 12 children with advanced HCC, sorafenib plus PLADO enabled surgical resection or transplantation in 43% of patients initially considered unresectable, with sustained remission in the majority of those who underwent surgery [8]. Our patient received four cycles of sorafenib-based therapy. Although not yet standard in children, sorafenib has demonstrated tumour shrinkage and improved resectability in small paediatric cohorts [9]. MRI after neoadjuvant chemotherapy showed a significant reduction in tumour size and no vascular invasion, allowing for definitive surgical resection.

Prognostically, complete (R0) surgical resection remains the strongest determinant of survival in paediatric hepatocellular carcinoma. In patients who undergo R0 resection, reported five-year overall survival ranges from 50% to 90%, depending on tumour subtype, stage, and presence of vascular invasion [7,10]. In contrast, children with unresectable or metastatic disease have markedly poorer outcomes, with five-year survival rates typically below 20%, underscoring the critical importance of achieving complete tumour clearance whenever feasible [7,8]. A ductal injury was diagnosed postoperatively due to increased biliary output from the abdominal drain, which prompted ERCP and biliary stenting. The patient went on to complete adjuvant chemotherapy with sorafenib. Recurrence rates after complete resection vary widely (25-50%) and typically occur within the first 12-24 months, underscoring the importance of close postoperative surveillance [7, 10]. In our patient, postoperative imaging and clinical assessments demonstrate that she remains disease-free at 11 months of follow-up, suggesting a positive recovery so far compared with what is normally reported in the literature. 

While complications are not uncommon in extensive hepatic resections, early recognition and intervention were critical to recovery. Complete resection remains the strongest predictor of survival in paediatric HCC [10]. Postoperative complications, if managed appropriately, do not significantly compromise long-term outcomes. Despite advancements, the prognosis of advanced-stage paediatric HCC remains poor. Prognosis is strongly correlated with resectability, absence of metastasis, and favourable biological markers [7,8,10].

## Conclusions

This case underscores the diagnostic complexity and treatment challenges of pediatric HCC, particularly in non-cirrhotic livers without identifiable risk factors. Histological ambiguity and the absence of classic biomarkers require careful multidisciplinary evaluation. While liver transplantation is the ideal curative approach for select patients with unresectable tumours, its unavailability in many low-resource settings necessitates alternative strategies. Our case demonstrates that neoadjuvant targeted therapy followed by aggressive surgical resection may represent a feasible curative alternative. This patient's outcome illustrates the importance of strategic multidisciplinary coordination and how treatment options can be tailored safely outside transplant-capable centres. However, we acknowledge that this is a single case and while instructive, this approach cannot be generalized without caution, as outcomes may differ based on tumour biology, resource availability, and patient-specific factors. 
